# Genome of Tomato Yellow Leaf Curl Virus (TYLCV) Obtained from Tomato Leaves in Kuwait

**DOI:** 10.1128/mra.01155-22

**Published:** 2023-06-26

**Authors:** Hanadi Al-Hashash, Abrar Akbar, Ebtisam Al-Ali

**Affiliations:** a Kuwait Institute for Scientific Research, Safat, Kuwait; DOE Joint Genome Institute

## Abstract

We report here the genome sequence of tomato yellow leaf curl virus (TYLCV) Wafra 19, isolated in Kuwait from symptomatic tomato plant leaves. The genome showed 98.42% identity to the AZ23-1 strain genome sequence.

## ANNOUNCEMENT

Whitefly-transmitted tomato yellow leaf curl virus (TYLCV) continues to be a major constraint to tomato production in Kuwait and in the region, reaching 100% loss in tomato plants (1). TYLCV was first characterized in Israel (2). In the last decade the virus has been identified in Iran, Oman, and Jordan (3). TYLCV is a single-stranded DNA (ssDNA) virus with a small genome that can infect tomato plants and cause several symptoms such as leaf curl, leaf deformation, silvering, upward cupping, yellowing, plant dwarfing, and fruit deformation (4). The virus belongs to the family *Geminiviridae* in the genus *Begomovirus* (5). Genome analysis can help in understanding the virulence behavior and can help in developing a virus-resistant tomato crop in the future.

The samples were collected from greenhouse-grown tomatoes in the Al-Wafrah area (28.63917 N, 47.93056 E). The viral DNA was obtained directly from tomato leaves showing symptoms using the cetyltrimethylammonium bromide (CTAB) method (6). The whole-genome sequencing was performed by Washington State University (Pullman, WA, USA) using the rolling-circle amplification (RCA) method and exoresistant random primers (TempliPhi 100 RCA kit). The RCA-derived reaction products were resolved by agarose gel electrophoresis, and a single product was selected based on size after agarose gel electrophoresis and sequenced using a Sanger sequencing platform (ABI system 3730). The genome was analyzed using the MUSCLE alignment tool (https://www.ebi.ac.uk/Tools/msa/muscle/) to verify the sequence by comparing it with other TYLCV genomes (JF451352, KR108214, KJ830842, and KJ830841) available in the GenBank database. Further verification included aligning the start and stop codons and identifying the proteins. The open reading frames (ORFs) were analyzed, and gene identity was determined using ORF Finder and BLAST in the NCBI GenBank database. The genome of TYLCV Wafra 19 was 2,781 bp long, and the G+C content was 41.3% with 5 predicted ORFs encoding c1 protein (replication-associated protein) (nucleotides [nt] 2615 to 1542), coat protein (nt 308 to 1084), c2 protein (transcriptional activation protein [TrAP]) (nt 1633 to 1226), c3 protein (replication enhancer protein [REn/C3]) (nt 1485 to 1081), and v2 protein (virus movement protein) (nt 106 to 498) (7). The TYLCV sequence was analyzed for identity and comparison using the Sequence Demarcation Tool (SDT), which showed high similarity to the AZ23-1 TYLCV strain genome sequence (98%) (8). Sequence analysis tools were run with default parameters unless otherwise specified.

This study will allow us to contribute to the knowledge of this virus in Kuwait and the region. In addition to that, such information can help in the development of certified virus-tested seeds to help in the disease management strategy.

### Data availability.

The full genome sequence of the TYLCV Wafra 19 strain has been deposited in GenBank under accession number OM691686. The version described in this paper is the first version, OM691686.1 (https://www.ncbi.nlm.nih.gov/nuccore/OM691686.1/). The Sequence Read Archive numbers are SRR24744412, SRR24744411, SRR24744410, SRR24744409, SRR24744408, and SRR23183369.
